# Fluid Shear Stress and Inner Curvature Remodeling of the Embryonic Heart. Choosing the Right Lane!

**DOI:** 10.1100/tsw.2008.42

**Published:** 2008-02-25

**Authors:** Beerend P. Hierck, Kim Van der Heiden, Christian Poelma, Jerry Westerweel, Robert E. Poelmann

**Affiliations:** ^1^ Department of Anatomy and Embryology, Leiden University Medical Center, The Netherlands; ^2^ Laboratory for Aero and Hydrodynamics - 3ME/P&E, Delft University of Technology, The Netherlands

**Keywords:** Biomechanics, blood flow, shear stress, cardiovascular development, endothelium, primary cilium

## Abstract

Cardiovascular development is directed or modulated by genetic and epigenetic factors. The latter include blood flow-related shear stress and blood pressure-related circumferential strain. This review focuses on shear stress and its effects on endothelial cells lining the inner surfaces of the heart and blood vessels. Flow characteristics of the embryonic blood, like velocity, viscosity and periodicity, are taken into account to describe the responses of endothelial cells to shear stress and the sensors for this friction force. The primary cilium, which is an integral part of the shear sensor, connects to the cytoskeletal microtubules and transmits information about the level and direction of blood flow into the endothelial cell. When the heart remodels from a more or less straight into a c-shaped tube the sharp curvature, in combination with the small vessel dimensions and high relative viscosity, directs the highest shear stress to the inner curvature of this pump. This proves to be an important epigenetic modulator of cardiac morphogenesis because when shear stress is experimentally altered inner curvature remodeling is affected which leads to the development of congenital cardiovascular anomalies. The best of both worlds, mechanics and biology, are used here to describe early cardiogenesis.

## INTRODUCTION

The heart develops as a primary heart tube from the splanchnic mesoderm as an endocardial vessel surrounded by a double layer of cardiomyocytes, in the midline of the embryo. Directly upon initiation of this primary tube, cardiac looping commences with a rightward shift of the outflow part of the heart. In this "c-looping" phase, the original dorsal side of the heart tube becomes the inner curvature[1,2]. The inner diameter of this curved vessel measures about 100–200 µm at this stage of development[3]. Peristaltic contraction of the tubular heart is initiated during early looping stages. Before connection of the embryonic vasculature to the vessels that develop in the yolk sac, blood flow through the heart is low and highly irregular. Upon connection to the extraembryonic vasculature, blood flow becomes regular and laminar by nature (see below). This review will focus on the role of blood flow on heart looping and inner curvature remodeling.

## BIOMECHANICAL CHARACTERISTICS OF BLOOD FLOW

Blood flow is an important nongenetic, or epigenetic, factor that modulates embryonic patterning, morphogenesis, and function. Biomechanical forces, exerted by blood flow and blood pressure, are registered by cells of the cardiovascular system that differentially respond to these functional cues. In an embryo, the forces include blood flow–induced shear stress and blood pressure–related stretch[4]. Both are cyclic by nature due to cardiac rhythm, and the latter primarily affects smooth muscle cells (SMC) and cardiomyocytes. Cyclic pressures cause circumferential stretch of the blood vessels to which SMC respond with alignment in the direction of the force, i.e., perpendicular to the long axis of the vessel. Whether myocardial orientation is also influenced by stretch forces is not clear yet. Wall shear stress (WSS), on which we will focus here, is the result of the friction of the flow at the wall. This friction, due to a finite viscosity or "stickiness" of the blood, is the reason that a pressure difference is always required for blood flow through a blood vessel. This driving force of the pressure drop is dissipated as the blood exerts a force on the vessel wall in the direction of the blood flow.

Shear stresses are present within the blood volume itself where they influence, for example, the rigidity of red blood cells, but also cause deformation or strain of endothelial cells at the lumen wall. Endothelial cells are especially responsive to WSS and respond to changes in shear forces with release of vasoactive substances and transcriptional activation of, for example, antithrombogenic pathways[5,6,7]. Shear stresses cannot be measured *in vivo*, but are usually derived using the dynamic viscosity (μ) and Newton's law of viscosity:

$$(\tau = \mu \frac{\delta u}{\delta x}).$$

To determine the value of the velocity (u) gradient (δu/δx) at the wall, it is commonly assumed that the flow exhibits a parabolic or Hagen-Poiseuille velocity profile. By combining this parabolic velocity profile and Newton's law, the following expression can be obtained for the WSS (τ):

$$\tau = \frac{4\mu Q}{\pi R^3},$$

where Q is the volumetric flow rate and R is the lumen radius of the blood vessel. In blood, viscosity depends on the shear rate that is illustrated by the "shear thinning" phenomenon, making blood essentially a non-Newtonian liquid. At low velocities, the biconcave red blood cells tend to pile together to form "rouleaux", which enormously increase viscosity. However, during early development, red blood cells are present as nucleated erythroblasts that do not have the capacity to form rouleaux, which results in a stable apparent viscosity that is largely independent of shear rates. Therefore, the use of Newton's law of viscosity is acceptable. There are spatial differences in viscosity that are important for shear-mediated endothelial function. The gradients in the (parabolic) blood velocity profile create lift forces that act on the red blood cells. This results in the fact that the concentration of red cells close to the wall is much lower than in the center of the vessel (Fåhraeus-Lindqvist effect). Therefore, WSS largely depends on plasma viscosity, rather than the viscosity of whole blood. In addition, the small dimensions of blood vessels in the embryo have another consequence with respect to blood flow characteristics: the Reynolds number (Re) and the Womersley number (α) are sufficiently low to facilitate laminar, nondisturbed, blood flow. Re represents the relation between inertial and viscous forces and is defined as:

$$\mathrm{Re} = \frac{2\rho u R}{\mu},$$

where ρ is the fluid density. Alpha describes the influence of instationary forces (i.e., pulsations) in relation to viscous forces and is defined as:

$$\alpha = R\left(\frac{\omega\rho}{\mu}\right)^{1/2},$$

where ω is the angular frequency (ω = 2πf, f is frequency). When Re < 2000, viscous forces dominate over inertial forces and, in the case of flow through circular geometries, the flow will be laminar. When Re < 1, inertia is completely negligible, a regime usually referred to as “creeping” or Stokes flow. In a chicken embryo at stage HH17, Re does not exceed unity and the laminar flow will closely resemble the theoretical Hagen-Poiseuille flow. A low Reynolds number furthermore indicates that the flow rapidly develops, i.e., after a change in vessel geometry the flow reaches the theoretical profile nearly instantly. In cases in which the Womersley number (α) is greater than unity, the flow profile will flatten compared to the “steady” parabolic profile. In the same chicken embryo α = 0.31, which again means that blood flow in the young embryo will resemble the theoretical case.

## SHEAR STRESS SENSING

Endothelial and endocardial cells require a sensor for shear stress in order to respond to changes in blood flow. Cells have been reported to be stimulated through the activation of, for example, integrins, G-protein receptors, tyrosine kinase receptors, or ion channels (reviewed by Lehoux and coworkers[8]). Especially, the interaction complex involving CD31/PECAM-1, VE-Cadherin, and VEGFR2/KDR/FLK-1 has been well documented[9]. In addition, the glycocalyx, a hydrated polysaccharide coat, has been proposed to be involved in shear stress sensing[10,11]. In contrast to a molecular sensor complex, an ultrastructural adaptation for mechanosensing, called a primary cilium, has been described[12,13]. Primary cilia are 1–5 µm, nonmotile, cellular protrusions with a 9+0 core of microtubules, and are the sensing counterparts of the motile (9+2) cilia or flagella that can be found on numerous cells types, like airway epithelium and sperm cells. The early embryonic determination of laterality, which also defines the direction of initial heart looping, is mediated through ciliated endodermal cells of the embryonic organizing center[14,15]. The clockwise rotation of motile cilia in the center of this area causes a leftward fluid flow that is sensed by adjacent cells with primary cilia through chemo- or mechanoreception (nicely reviewed by Bisgrove and Yost[16]). This results in a rise in intracellular $Ca^{2+}$ and activation of the Nodal signaling program on the left side of the organizing center, which forms the basis of asymmetric development. Interestingly, many targeting models for ciliary proteins present with *situs inversus*, which includes randomization of cardiac looping[17,18]. Left-right asymmetry determination and the role of ciliary proteins is highly conserved among species[19], emphasizing its importance.

Endothelial and endocardial cells can also be ciliated[20,21,22]. During embryogenesis, ciliated endothelium is abundantly present in the heart in areas of low and oscillatory, i.e., with flow reversals, WSS[21]. In the adult, endothelium is ciliated in proatherogenic areas with low and disturbed shear stress, irrespective of plaque formation[22]. The mechanism by which the primary cilium is involved in fluid shear sensing is probably dual. In endothelial cells, transmembrane proteins polycystin-1 and -2 localize to the cilium, and bending of the cilium induces an acute calcium transient and nitric oxide release[23]. This is mediated through activation of polycystin-2, which is a calcium channel and is involved in polycystic kidney diseases[24]. A prolonged response to fluid shear stress is mediated by the cilium, which functions as a lever to facilitate and amplify conformational changes of the cytoskeleton[25]. We recently demonstrated that cilia, in fact, sensitize endothelial cells for shear stress, a process that largely depends on cytoskeletal microtubules[26].

## SHEAR STRESS AND CARDIOVASCULAR DEVELOPMENT

What is the role of shear stress in the morphogenesis and differentiation of the cardiovascular system? Emerging evidence indicates a delicate balance between genetic determination and hemodynamic modulation. A good example for that is the arterial, venous, or lymphatic identity of developing blood vessels in the embryo. Zebrafish studies have elegantly shown that the initial identity of vessels is genetically predetermined[27]. However, hemodynamic forces can subsequently alter or modify this phenotype[28,29].

Endothelial cells are particularly sensitive to fluid shear stress. Microarray screens have identified numerous genes that were either up- or down-regulated by shear forces. The zinc finger transcription factor Krüppel-Like Factor 2 (*KLF2*) appears to play a central role in the regulation of shear-mediated gene expression in endothelial and endocardial cells. *KLF2* is induced by high shear stress[30] through the *MEK5-ERK5-MEF2* and *PI3K-NCL* signaling pathways[31,32,33]. It plays an important role in vascular tone regulation[5], partly through activation of endothelial nitric oxide synthase (*eNOS*/*NOS3*) and repression of endothelin-1 (*EDN1*/*ET1*). The latter is vasoconstrictive, whereas NO produced by NOS3 is a vasodilator. In the embryonic chicken heart and vasculature, *KLF2* is specifically expressed by endocardial cells with high expression in the atrioventricular canal, the inner curvature, and the outflow tract (Fig. 1; [34]). *KLF2* and *ET1* expression patterns partly overlap in the chicken embryonic heart up to stage HH21, whereas later on, expression becomes mutually exclusive. *NOS3* expression overlaps, but also extends that of *KLF2*[35,36].

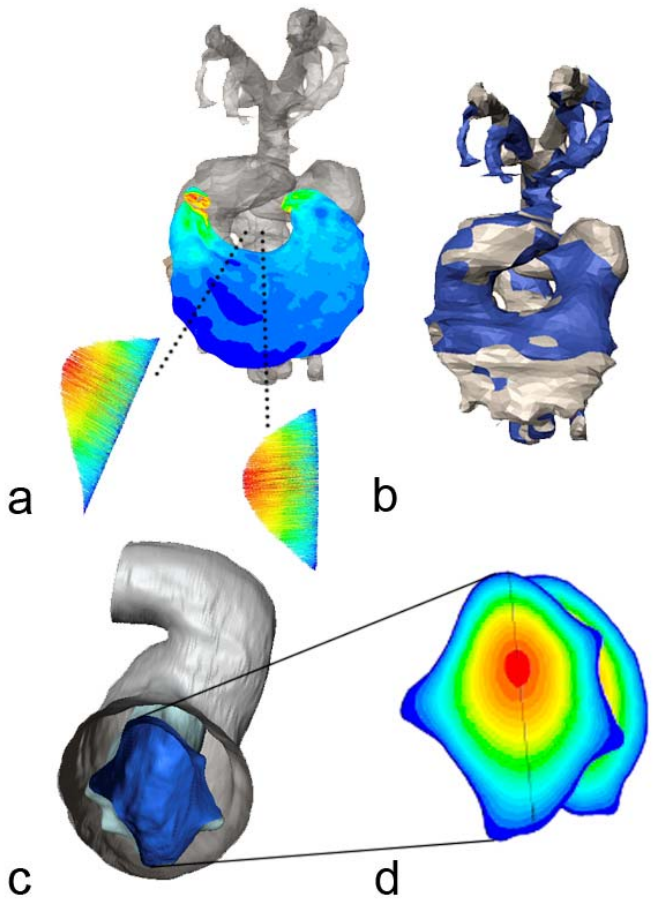

**FIGURE 1.** Shear stress distribution in the developing heart. Computational fluid dynamics analysis of the predicted shear stress distribution in the HH17 chicken embryonic heart (a) and delineation of the endocardial mRNA expression of the shear response marker *KLF2* (b, blue). Both patterns are superimposed on the 3D geometry of the heart and vessels (grey). Note that the predicted shear levels in the inner curvature are higher (light blue in a) compared to the outer curvature (dark blue in a), and that *KLF2* mRNA expression is restricted to the high shear areas. The insets in (a) represent the velocity profiles in the dashed areas and show the same inward shift in peak velocity to the inner curvature. A cross-sectional profile of the geometry and the velocity profile is shown in (c) and (d), respectively. Figures a and b are adapted from Hierck et al.[53].

Expression of the high shear marker *KLF2* in the inner curvature of the heart was somewhat surprising as, intuitively, one would expect shear stress to be high in the outer curvature. To understand the fluid behavior in the curved tubular heart, the Dean number (Dn) should be considered. Dn describes the relation between the curvature of the vessel (ratio of vessel radius R and radius of the curvature $R_c$) and the Reynolds number as:

$$Dn = \mathrm{Re}\left(\frac{R}{R_c}\right)^{1/2},$$

and expresses the relevance of the centrifugal forces due to curvature compared to the viscous forces. With large values of Dn a secondary flow pattern occurs, which leads to a shift of the maximum flow velocity toward the outer wall of the curvature. However, the strong curvature of the embryonic heart, combined with a small diameter (100–200 μm), results in a small Dn and a shift of the maximum velocity toward the inner vessel wall[37]. Numerically, this has been demonstrated elegantly by Wang and Bassingthwaighte[38]. Experimentally, we have confirmed this phenomenon in the chicken embryonic heart by high-resolution microparticle image velocimetry (PIV) technology[39], and showed a peak velocity of 26 mm/sec in the conotruncal region of a HH16 chicken heart, which corresponds to a peak shear stress of 5 Pa (50 dyne/cm$^2$), with highest shear in the inner curvature (see Fig. 2). PIV is an optical technique that is based on the displacement of particles in a flow field. Cross-correlation is used to resolve the particle displacement in two consecutive pictures, which results in a high-resolution vector plot. A detailed description of this technique in relation to other measurements methods for blood flow is provided by Vennemann and colleagues[40]. Similar techniques, utilizing fluorescent red blood cells, have been used to demonstrate and calculate hemodynamics in murine and zebrafish embryos[41,42,43,44]. By seeding the blood volume with small (0.5–1 μm) fluorescent particles, we were able to penetrate the cell-free zone, described above, to estimate reliably the actual WSS that is experienced by the endocardial cells. We and others[45,46] have confirmed these findings by modeling blood flow through the natural geometry of a HH14 heart (see Fig. 2). This geometry was derived from a 3D AMIRA (Mercury Computer Systems) reconstruction of a confocal stack through a fully dilated chicken embryonic heart, of which the endothelium was fluorescently stained with *Sambucus nigra* lectin (SNA-FITC, Vector Laboratories). Subsequently, this geometry was fed into the Fluent computational fluid dynamics (CFD) package, and the patterning of flow distribution and shear stress patterning was analyzed showing high shear levels in the inner, relative to the outer, curvature. Possible consequences of these findings are discussed below.

## VENOUS CLIP MODEL

By disturbing hemodynamics in the developing embryo, cardiovascular development can be experimentally altered. Among these models are the left atrial banding model[47], conotruncal banding[48], and the venous clip model[49,50,51]. The latter is a chicken embryo model in which blood flow through the heart is experimentally altered by transient interference with the venous inflow. This causes a series of events, centered along the inner curvature of the heart, which lead to developmental anomalies that involve cardiac looping. In this model, the right lateral vitelline vein, which drains the right side of the yolk sac vasculature, is permanently ligated. Blood from this vessel then reroutes through the pre-existing caudal capillary plexus to the left lateral vitelline vein where it drains into the sinus venosus through the omphalomesenteric vein. Intracardiac blood flow patterning changes with an inward shift, i.e., in the direction of the inner curvature. The embryo responds with a rapid, but transient, change in functional parameters[52], which are back to physiological levels within 12 h. The redistribution of blood in the heart and the functional adaptations lead to a change in the expression patterns of shear stress–related genes. The high shear stress marker *KLF2* is elevated in the endocardium lining the inner

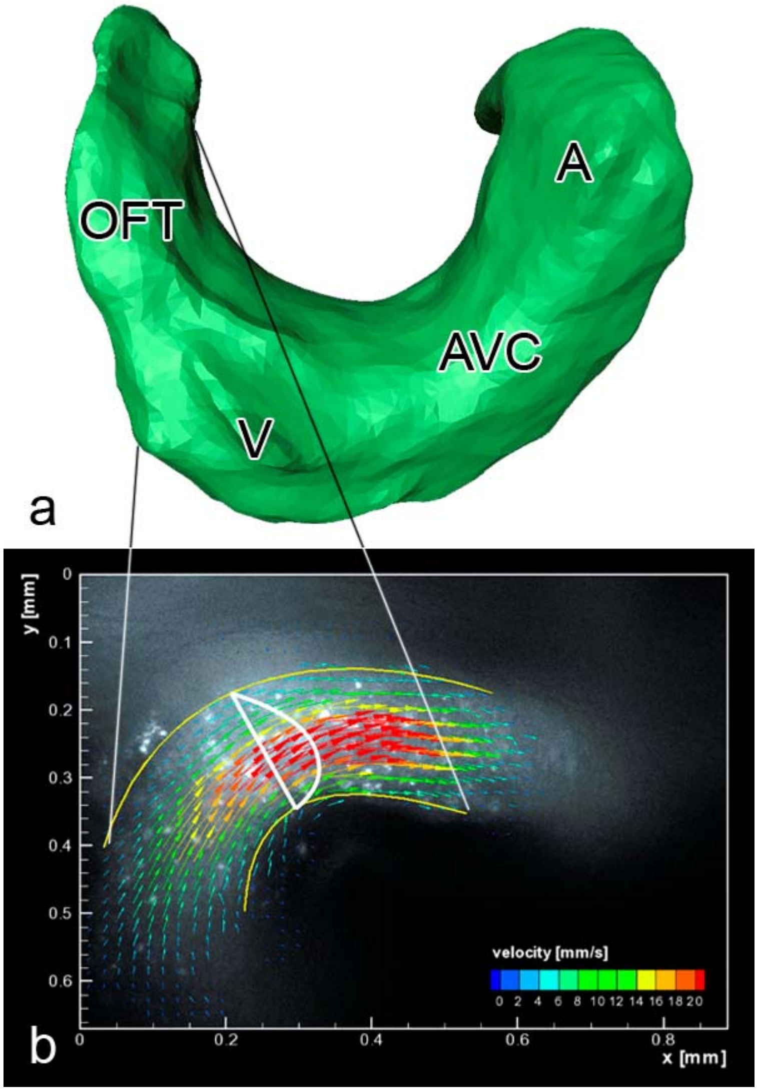

**FIGURE 2.** Geometry and measured velocity profile of the heart at HH16. The endocardium of the HH16 chicken embryonic heart was fluorescently labeled and analyzed by CLSM. (a) The natural geometry of the lumen in full dilatation was deduced by subsequent 3D reconstruction. (b) Systolic blood flow in the outflow tract (OFT) area of a living HH16 chicken embryo was quantified by µPIV analysis. Note that the measured velocity profile is asymmetric with a shift of maximum velocity to the inner curvature of the heart. A = atrium, AVC = atrioventricular canal, V = ventricle. Fig. b is adapted from Vennemann et al.[39].

curvature of the heart and *ET1* mRNA is down-regulated in this area. These expression changes were apparent within 3 h after ligation, which indicates that changes were due to experimentally induced hemodynamic alterations rather than due to abnormal morphology (reviewed in [35,53]). After 1 to 2 days, functional parameters of the heart are changed permanently in the experimental animals[54,55,56,57], leading to heart malformations such as double outlet of the right ventricle, ventricular septal defects, and atrioventricular and semilunar valve anomalies, which were all related to abnormal cardiac looping[49,50]. Interestingly, these looping disturbances are most probably not related to altered expression or signaling through ET1, since recent data show that infusion of this growth factor or blockage of its receptors during looping stages does not phenocopy the looping-related anomalies as evoked in the venous clip model[58].

## INNER CURVATURE REMODELING

As described above, nodal flow is important in left-right determination, mediated through signaling in which nodal-related ligands of the transforming growth factor-beta superfamily play a central role[59], and concomitant looping of the primary heart tube. Early experimental data (reviewed by Taber[2]) and more recent zebrafish data using *sih* and *cfk* mutants without early blood flow[60] indicate that genetic factors, rather than blood flow–mediated shear stresses, are the driving force for the direction of looping in the first phase of heart development. During the second phase of heart looping, in which the cardiac tube remodels from a "c" into an "s" shape, blood flow is initiated and WSS becomes an important functional mediator. Compaction of the inner curvature tissues is necessary for subsequent wedging of the right ventricular inflow tract to the right and of the outflow tract to the left. Disturbed or delayed wedging generally leads to septation problems such as double inlet of the left ventricle (DILV) or atrioventricular septal defects (AVSD) in the ventricular inflow region, or double outlet of the right ventricle (DORV) or transposition of the great arteries (TGA) in the outflow region (elegantly reviewed by Ramsdell[17]). Ventricular septal defects (VSDs) usually accompany these anomalies. As a result of cardiac anomalies, blood flow through the pharyngeal arch arteries is altered, rendering them susceptible for abnormal remodeling and the development of congenital vascular malformations[61,62].

It is now generally accepted that mechanical forces greatly influence inner curvature remodeling. Linask and Vanauker described the distribution and role of the nonmuscle myosin II cytoskeletal element in heart looping[63]. In addition Taber reviewed the biophysical mechanism of "c" looping[2]. These studies largely focus on strain due to contraction forces, which is obvious because these are much higher than shear forces. However, endothelial cells are much more sensitive for shear stress compared with stretch[26] and changes in shear patterning rapidly result in local changes in shear-related endothelial gene expression, including the expression of important growth factors like endothelin-1[45]. Remodeling or compaction of the inner curvature of the looping heart tube includes the myocardium as well as the endocardial (atrioventricular) cushions. Looping coincides with epithelial to mesenchymal transformation by which endocardial cells transform and fill the cushions, a process that is affected by changing the shear stress in the inner curvature in the venous clip model[64]. Loading of the cushions with cells results in a dramatic nonlinear increase in cushion stiffness[65]. Because of this nonlinearity, shear-induced deformation, or "prestretch", increases the modulus, rendering endocardial and cushion mesenchymal cells more sensitive for deformation changes[66] even in the absence of endocardial primary cilia in the cushion area[21]. Since WSS differentiates between inner and outer curvatures, as discussed above, we propose an important signaling role for this biomechanical epigenetic factor in inner curvature remodeling. This could mediate, for example, the myocardial tonus and contraction[63], the transition in cardiac contraction from peristaltic to an apex-to-base profile[65], influx of epicardial-derived cells in the inner curvature myocardium[67], and endothelin signaling[35,58]. Balance is the key word in this phase of heart development. Balance between genetic and epigenetic factors, balance between various signaling pathways, balance between shear and stretch forces, and balance between inner and outer curvature remodeling.

## ACKNOWLEDGMENTS

We like to thank Martin Baiker and Mathieu Pourquie (Department Aero and Hydrodynamics, Delft University of Technology, The Netherlands) for the 3D modeling and CFD analyses, and Jan Lens for the artwork.
